# *Panax Notoginseng Saponins* Protect Cardiac Myocytes Against Endoplasmic Reticulum Stress and Associated Apoptosis Through Mediation of Intracellular Calcium Homeostasis

**DOI:** 10.3389/fphar.2019.01013

**Published:** 2019-09-20

**Authors:** Jun Chen, Rui Xue, Li Li, Li Li Xiao, Jiahong Shangguan, Wenjing Zhang, Xueyang Bai, Gangqiong Liu, Ling Li

**Affiliations:** ^1^Vasculocardiology Department, The First Affiliated Hospital of Zhengzhou University, Zhengzhou, China; ^2^Medical Research Center, The First Affiliated Hospital of Zhengzhou University, Zhengzhou, China

**Keywords:** *Panax notoginseng* saponins, endoplasmic reticulum stress, apoptosis, intracellular calcium homeostasis, ryanodine receptor

## Abstract

Endoplasmic reticulum (ER) stress has been demonstrated to play important roles in the pathogenesis of various cardiovascular diseases. The ER stress pathway is therefore a promising therapeutic target in cardiovascular disease. Although *Panax notoginseng* saponins (PNS) are one of the patent medicines that are traditionally used to treat cardiovascular disorders, their effects on ER stress in cardiac myocytes remain unexploited so far. This study investigates the effects of PNS on ER stress and its associated cell apoptosis along with the related mechanism in cardiac myocytes. PNS compounds were identified *via* high-performance liquid chromatograph (HPLC) assay. PNS-pretreated H9c2 cells, HL-1 cells, and primary cultured neonatal rat cardiomyocytes were stimulated with thapsigargin (TG) to induce ER stress response and apoptosis. ER stress response was tested by immunofluorescence or immunoblot of the ER protein chaperones—calnexin, binding immunoglobulin protein (BiP) and the C/EBP homologous protein (CHOP). Cell viability was tested by methyl thiazolyl tetrazolium (MTT) assay. Cell apoptosis was detected by immunoblot of Cleaved caspase-3 and flow cytometry analysis of Annexin V/propidium iodide (PI) staining. Cytosolic, mitochondrial, and ER calcium dynamics were investigated by calcium imaging. Moreover, a ryanodine receptor type-2 (RyR_2_) overexpression stable cell line was generated to verify the mechanism of RyR_2_ involved in PNS in the inhibition of ER stress and cell apoptosis. We demonstrate here that PNS protected cardiac myocytes from ER stress response and associated cell death in a concentration-dependent manner. Importantly, PNS reduced the elevation of cytosolic calcium, mitochondria calcium, as well as ER calcium in response to either TG or histamine treatment. PNS protection in ER stress was regulated by RyR_2_ expression. In summary, PNS protection against TG-induced ER stress response and its associated cell apoptosis in cardiac myocytes is calcium dependent. Through the regulation of ER calcium release mediated by RyR_2_, a novel mechanism for PNS in the prevention of cardiovascular diseases is thereby identified.

## Introduction

The endoplasmic reticulum (ER) is a multifunctional organelle essential for the synthesis, folding, and processing of secretory and transmembrane proteins. Pathological stimuli that disrupt the ER homeostasis resulting in an accumulation of misfolded and unfolded proteins are known as ER stress. ER stress evokes a protective and compensatory mechanism referred to as the unfolded protein response (UPR), which serves multiple functions, including the assistance of protein folding *via* the upregulated ER protein chaperones and the enhanced degradation of misfolded proteins *via* the upregulation of molecules involved in the ER-associated protein degradation (ERAD) pathway (Brewer et al., 1997; Friedlander et al., 2000; Hampton, 2000). However, if the ER stress is too excessive to re-establish the ER function, cell dysfunction and subsequent cellular death may occur. Thapsigargin (TG) is a highly selective inhibitor of sarco/endoplasmic reticulum (SR/ER) Ca^2+^-ATPase (SERCA), which inhibits Ca^2+^ transfer from ER to cytosol, thereby elevating intracellular calcium concentration (Thastrup et al., 1990). Furthermore, TG disturbs the calcium homeostasis and leads to protein misfolding, causing the accumulated misfolded/unfolded proteins to induce ER stress. In addition, prolonged TG treatment initiates the intrinsic apoptotic pathway by permeabilizing the mitochondrial membrane, releasing cytochrome c and apoptosis inducing factor (AIF) to cytosol, resulting in apoptosome formation, and thus leading to the activation of caspase-3 (Rao et al., 2002).

ER stress and associated apoptosis have been demonstrated to play important roles in the pathogenesis of various cardiovascular diseases, such as cardiac hypertrophy, heart failure (HF), ischemic heart disease, and atherosclerosis (Kassan et al., 2012; Padilla and Jenkins, 2013; Shinozaki et al., 2013). ER stress–induced abnormality of the intracellular Ca^2+^ stores and the SR Ca^2+^ release in the heart play prominent negative roles in cardiac contractile activation and relaxation (Eisner et al., 2000; Bers, 2002). Alterations in the sensitivity of ryanodine receptor (RyR) to Ca^2+^ release activation have been involved in various diseases such as malignant hyperthermia and HF (Loke and MacLennan, 1998; Marx et al., 2000; Kushnir et al., 2018). Diastolic SR Ca^2+^ leak decreased SR Ca^2+^ load and reduced contractility along with cardiac output (Shan et al., 2010). Thus, chronic SR Ca^2+^ leak *via* ryanodine receptor type-2 (RyR_2_) channels causes mitochondrial Ca^2+^ overload and metabolic dysfunction in hearts (Santulli et al., 2015).

Increasing evidence suggests a promising therapeutic strategy by targeting the ER stress pathways with natural products (Choy et al., 2018; Hu et al., 2018; Xu et al., 2018). *Panax notoginseng* saponins (PNS), mainly derived from *Panax notoginseng*, are patent medicines that are commonly used as treatment for cardiovascular disorders, such as ischemia reperfusion–induced cognitive impairments, atherosclerosis, platelet aggregation, reperfusion arrhythmias, strokes, coronary artery disease (CAD), and HF (Ma and Xiao, 1998; Zheng et al., 2008; Yuan et al., 2011). To date, 40 ginsenoside components have been identified and quantified from different parts of *Panax* (Kim, 2018). Several studies have detailed the antioxidant, anti-inflammation, and anti-apoptosis effects of PNS (Wang et al., 2011; Huang et al., 2017; Zhou et al., 2018); however, the prohibitive effects of PNS related to ER stress have not been reported.

Therefore, here in this study, we focused on PNS protection in TG-induced ER stress response and associated cell apoptosis in cardiac myocytes, especially in the regulation of intracellular Ca^2+^ homeostasis. We mainly examined the effects of PNS on TG-induced alternations of ER network morphology, expression of UPR-involved proteins chaperone binding immunoglobulin protein (BiP, also known as the glucose-regulated protein 78/Grp78) and the C/EBP homologous protein (CHOP, also known as growth arrest- and DNA damage-inducible gene 153/GADD153), cell viability, expression of apoptotic gene caspase-3, as well as the intracellular calcium homeostasis and associated calcium handling proteins. The results revealed a novel mechanism of PNS in the protection of cardiac myocyte survival upon cell stress.

## Materials and Methods

### Chemicals

PNS was purchased from Kunming Pharmaceutical Corporation (Yunnan, China; patent no. ZL96101652.3) with the major effective constituents including notoginsenoside R1 9.8% (v/v), ginsenoside Rb1 32.3% (v/v), ginsenoside Rg1 35.3% (v/v), ginsenoside Re 4.0% (v/v), and ginsenoside Rd 4.9% (v/v) and the total pharmaceutical concentration of ∼90% (v/v). TG (content ≥ 98%) was purchased from Sigma (T9033, Sigma-Aldrich, USA). Bapta-acetoxymethyl (AM) (content ≥95%) was from Sigma (A1076, Sigma-Aldrich, USA), and ionomycin (content ≥ 98%) was from Sigma (I9657, Sigma-Aldrich, USA).

### High-Performance Liquid Chromatograph

High-performance liquid chromatograph (HPLC) was performed using Agilent 1200 series (Agilent Technologies, USA). The LC column used was Chromolith Performance RP-18, 100 × 4.6 mm, 2 μm (1.02129, Sigma-Aldrich, USA). The mobile phase consisted of water (A) and acetonitrile (B) with the following gradient protocol: 0 min, 16% B, 3 ml/min; 3 min, 16% B, 3 ml/min; 10 min, 19% B, 3 ml/min; 11 min, 19% B, 2.5 ml/min; and 20 min, 38% B, 2.5 ml/min. The column oven was set at 30°C. The injection volume was 10 μl. Ultraviolet (UV) absorption was measured at 203 nm.

### Cell Lines Culture and Treatments

Rat cardiomyoblast H9c2 cell line was obtained from the American Type Culture Collection (ATCC# CRL-1446^TM^, ATCC, USA). H9c2 cells were grown in dulbecco's modified eagle's medium (DMEM) containing 10% fetal bovine serum (FBS), supplemented with l-glutamine (2 mM), penicillin (100 U/ml), and streptomycin (100 µg/ml), in a humidified atmosphere containing 5% CO_2_. Cardiac muscle HL-1 cell line was from Sigma (SCC065, Sigma-Aldrich, USA). HL-1 cells were grown in gelatin/fibronectin extracellular matrix (ECM)-coated dishes (G9391 and F1141, Sigma-Aldrich, USA) and cultured in Claycomb medium (51800C, Sigma-Aldrich, USA) containing 10% FBS (TMS-016B, Sigma-Aldrich, USA), supplemented with l-glutamine (2 mM), norepinephrine (0.1 mM, A0937, Sigma-Aldrich, USA), and penicillin–streptomycin (100 U/ml–100 µg/ml, P4333, Sigma-Aldrich, USA).

For PNS pretreatment, cells were starved overnight and incubated with fresh media containing various concentrations of PNS for 12 h. Cells were subsequently treated with 1 μM TG in media containing various concentrations of PNS for 12 h to induce ER stress or 24 h to induce apoptosis, respectively.

### Primary Culture of Neonatal Rat Cardiomyocytes and Purification Identification

Cardiomyocytes were obtained by dissociating hearts of neonatal Sprague–Dawley rats (1–3 days old). The experimental protocol for animals was approved by the Ethics Committee for Scientific Research and Clinical Trails of the Affiliated Hospital of Zhengzhou University. In detail, cardiomyocytes were isolated enzymatically using a neonatal cardiomyocyte isolation system (Worthington Biochemical, USA). Then a “pre-plating” step was introduced to extract fibroblasts and endothelial cells from the cardiomyocytes. The cardiomyocyte-enriched supernatant was then seeded onto cell culture plates pre-coated with 10 μg/ml of fibronectin and cultured in dulbecco's modified eagle's medium/ham's nutrient mixture F-12 (DMEM/F12) media containing 10 mM hydroxyethyl piperazineetha nesulfonic acid (HEPES), 10% FBS, penicillin–streptomycin (100 U/ml–100 µg/ml), and 0.1 μmol/ml bromodeoxyuridine (BrdU) to inhibit non-myocyte cell proliferation. Cardiomyocyte purification was identified by immunofluorescence assay of the anti-*α*-actin antibody. The cells were treated with PNS or TG as indicated above after 72 h of tissue culture.

### Cell Viability Assay

The cell viability was tested by the methyl thiazolyl tetrazolium (MTT) assay (M2128, Sigma-Aldrich, USA). H9c2 cells were seeded in a 96-well plate with 5,000 cells per well for 48 h. Cells were then treated by various concentrations of PNS with or without 1 μM TG stimulation for 24 h. Twenty microliters of MTT (5 mg/ml) was added to each well for 4 h and then replaced by 150 µl dimethyl sulfoxide (DMSO). The optical density of the plate was measured at 570 nm using a multilabel microplate reader (VICTOR^TM^ X4, PerkinElmer, Inc., USA). The cell viability of the untreated control was considered as 100%.

### Immunofluorescence Assays

For immunofluorescence assays, cells were fixed with 4% paraformaldehyde (PFA) and permeabilized with 0.2% Triton. After blocking with 1% bovine serum albumin (BSA), cells were then incubated with the primary anti-*α*-actin antibody (ab5694, 1:500, abcam, USA), anti-calnexin antibody (AF18, 1:500, Thermo Fisher Scientific, USA), anti-Tom20 antibody (sc-17764, 1:500, Santa Cruz Biotechnology Inc., USA), anti-derlin (sc-390289, 1:500, Santa Cruz Biotechnology Inc., USA), or anti-green fluorescent protein (GFP) (PA5-22688, 1:1,000, Thermo Fisher Scientific, USA) for 60 min at 37°C, respectively, followed by secondary antibodies, Alexa 488 immunoglobulin G (IgG) (A32723, 1:1,000) or Alexa 568 IgG (A11011, 1:1,000) (Thermo Fisher Scientific, USA), in a dark chamber. Cells were examined using a ZEISS LSM510 META laser-scanning confocal microscope (Carl Zeiss, Germany). Fluorescence images were acquired by a Plan-Neofluar 20×/0.40 LD or Plan-Apochromat 63×/1.40 Oil objective with either 488 nm laser excitation [520 band pass (BP) emission] or 543 nm laser excitation [570 long pass (LP) emission].

### Flow Cytometry Analysis

Cell labeling for Annexin V/propidium iodide (PI) was performed according to the kit’s instructions (V13242, Thermo Fisher Scientific, USA). Cells were analyzed by BD LSRFortessa cell analyzer (BD Bioscience, USA) using dual-wavelength excitation at 488 and 568 nm and detection at 515–565 nm and 600–670 nm for fluorescence detection.

### Cytosolic and Mitochondria Ca^2+^ Measurements

Cells were plated in a glass-bottom petri dish, 35 mm, for 24 h (No. 1.5, MatTeK Corporation, USA). Cells were incubated in calcium imaging solution (in mM: 145 NaCl, 5.4 KCl, 0.5 MgCl_2_, 1.2 CaCl_2_, 5 HEPES, 5.5 glucose, 0.3 NaH_2_PO_4_, pH 7.4) containing 5 μM Fura-2 AM (F1221, Thermo Fisher Scientific, USA) or 5 μM Rhod-2 AM (R1245MP, Thermo Fisher Scientific, USA), supplied with 0.02% Pluronic F-127 (P3000MP, Thermo Fisher Scientific, USA) to help disperse AM ester for 45 min at 37°C, respectively. Cells were illuminated at alternating excitation wavelengths of 340 and 380 nm for Fura-2 or a monochromatic excitation wavelength of 540 nm for Rhod-2 in an Epi-fluorescence Eclipse Ti microscope with a Plan-Fluor 40×/1.3 Oil objective (Nikon, Japan). The emitted fluorescence was recorded at 510 nm for Fura-2 or 575 nm for Rhod-2 with an Andor Zyla scientific complementary metal oxide semiconductor (sCMOS) camera (Oxford Instruments, UK). Exposure time was typically 100–200 ms, and images were collected every 10–20 s. Images were analyzed using MetaFluor software (Universal Imaging Corporation, USA). Fluorescence images were background-corrected, and cells with similar Fura-2 or Rhod-2 fluorescence intensity were analyzed. Nuclear signal was excluded when quantifying the Rhod-2 fluorescence signals.

### ER Ca^2+^ Measurements

Cells were plated in a glass-bottom petri dish, 35 mm, for 24 h (No. 1.5, MatTeK Corporation, USA) and transiently transfected with the fluorescence resonance energy transfer (FRET)–based ER-targeted cameleon (D1ER) (Palmer et al., 2004). Cells were incubated in Ca^2+^ imaging solution (in mM: 145 NaCl, 5.4 KCl, 0.5 MgCl_2_, 1.2 CaCl_2_, 5 HEPES, 5.5 glucose, 0.3 NaH_2_PO_4_, pH 7.4) and imaged by an Epi-fluorescence Eclipse Ti microscope with a Plan-Fluor 40×/1.3 Oil objective (Nikon, Japan). Emission ratio imaging of the cameleon was accomplished by excitation wavelengths of 425 nm with a dichroic mirror at 515 nm and two emission filters [475 nm for enhanced cyan fluorescent protein (ECFP) and 535 nm for citrine-yellow fluorescent protein (YFP)] (Chroma Technology Corporation, USA). Changes in ER calcium were expressed as the FRET-to-CFP emission ratio. Exposure times were typically 100–200 ms, and images were collected every 10–20 s. Images were analyzed using MetaFluor software (Universal Imaging Corporation, USA). Fluorescence images were background-corrected, and cells with similar cameleon expression were analyzed.

### Western Blot Analyses

Protein (100–120 μg of total protein per lane) was separated through sodium dodecyl sulfate-polyacrylamide gel electrophoresis (SDS-PAGE) gels of 5% (for RyR_2_), 10% (for SERCA_2_ or BiP), and 12% (for CHOP, Cleaved caspase-3, or β-actin) and transferred to polyvinylidene difluoride (PVDF) membranes. The PVDF membranes were probed with anti-SERCA_2_ (sc-376235, 1:500, Santa Cruz Biotechnology Inc., USA), anti-RyR_2_ (ab2868, 1:500, abcam, USA), anti-BiP (ab21685, 1:1,000, abcam, USA), anti-CHOP (sc-7351, 1:500, Santa Cruz Biotechnology Inc., USA), or anti–Cleaved caspase-3 (#9664S, 1:800, Cell Signaling Technology, USA), followed by appropriate horseradish peroxidase (HRP)-conjugated secondary antibodies. The β-actin (A5316, 1:10,000, Sigma-Aldrich, USA) gene was used as the internal standard for normalization of the protein samples. Chemiluminescence was revealed using Pierce^TM^ enhanced chemiluminescence (ECL) Western Blotting Substrate (32106, Thermo Fisher Scientific, USA) and densitometry performed using Quantity One 1-D software (Bio-Rad Laboratories, USA).

### Inducible RyR_2_ Gene Overexpression Cell Lines

The coding sequence for mouse RyR_2_ (NM_023868.2) was amplified by polymerase chain reaction (PCR) using complementary deoxyribonucleic acid (cDNA) from a mouse ventricle as the template and cloned into the expression vector of pcDNA3.1. Stable cells carrying GFP-tagged RyR_2_ were generated by transfection with RyR_2_-multifunctional GFP (mfGFP) in pcDNA3.1(+) and selected with 1,600 μg/ml G-418 (30-234-CI, Corning, USA). HL-1 cells stably expressing either the empty pcDNA3.1(−) vector [non-targeted control (NTC)] or pcDNA3.1 vector containing RyR_2_ (RyR_2_-mfGFP) were cultured as described previously (Liu et al., 2002). The expression level of RyR_2_ was identified by immunofluorescence and immunoblot analyses.

### Statistical Analyses

Data are presented as mean ± the standard error of the mean (SEM). Differences between means were determined using the one-way analysis of variance (ANOVA) for group-paired observations. Differences were considered statistically significant when *P* < 0.05.

## Results

### PNS Compound Identification

**Figure 1:**
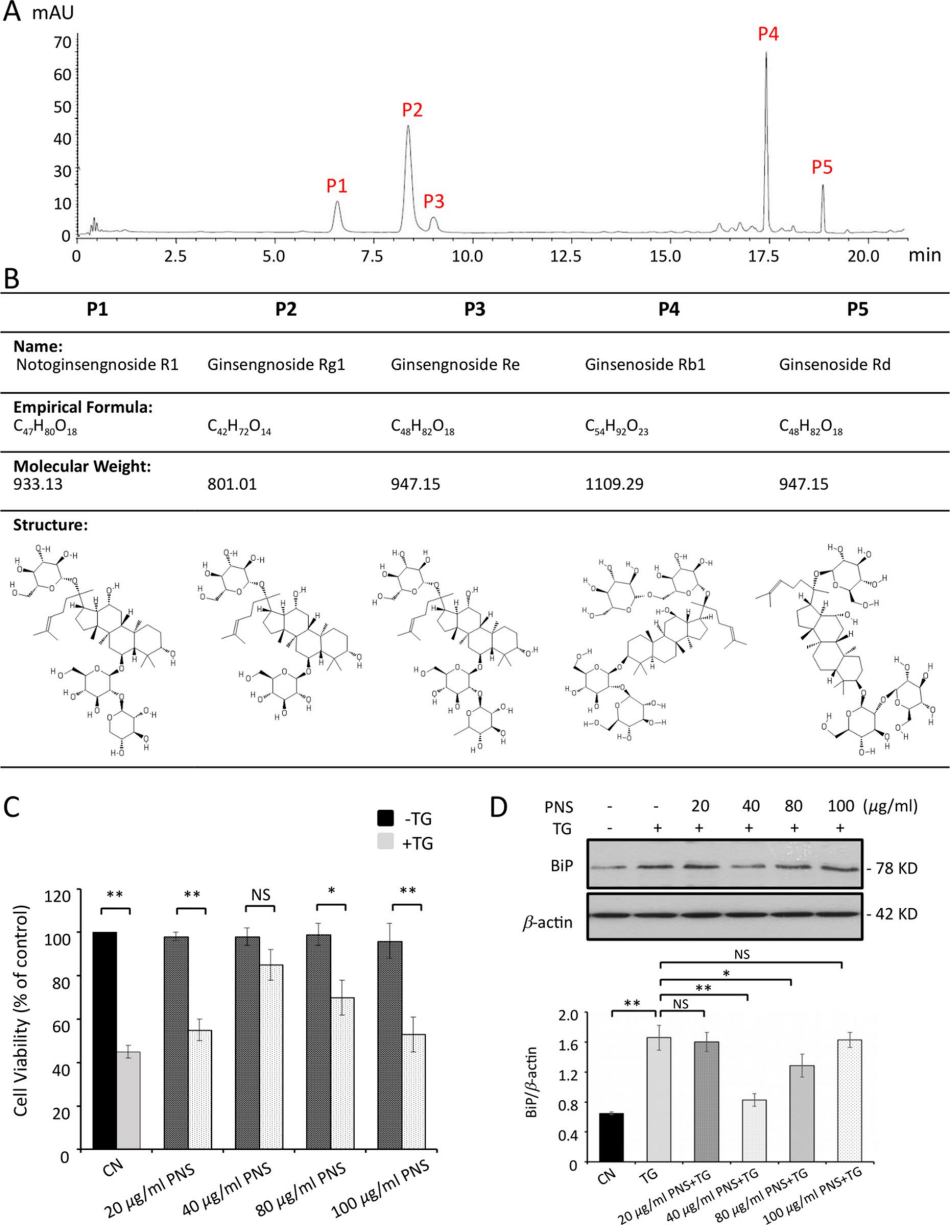
PNS compound identification and cell viability test. **(A)** HPLC profiles of PNS compounds. P1: notoginsenoside R1; P2: ginsenoside Rg1; P3: ginsenoside Re; P4: ginsenoside Rb1, and P5: ginsenoside Rd. **(B)** PNS compound identification: formula, molecular weight, and structure. **(C)** Cell viability tests of H9c2 cells treated with various concentrations of PNS (0, 20, 40, 80, 100 μg/ml) either with or without 1 μM TG (dark, −TG group; grey, +TG group). Bar graph shows percentage of viability compared with the untreated cells. **(D)** H9c2 cells treated as indicated in C were immunoblotted with antibodies to BiP and β-actin. Bands were quantified relative to β-actin by densitometry. (Mean ± SEM; NS, not significant; *P < 0.05, **P < 0.01 relative to CN group or indicated group.) PNS, *Panax notoginseng* saponin; HPLC, high-performance liquid chromatograph; TG, thapsigargin; BiP, binding immunoglobulin protein; SEM, the standard error of the mean; CN, control.

The compounds of freeze-dried PNS powder were identified by HPLC analysis at an absorbance of 203 nm. Five saponins were completely separated within 20 min without significant interference (**Figure 1A**). The retention times were 6.7, 8.4, 9.2, 17.5, and 18.8 min for P1: notoginsenoside R1, P2: ginsenoside Rg1, P3: ginsenoside Re, P4: ginsenoside Rb1, and P5: ginsenoside Rd, respectively. The formula, molecular weight, as well as the structure for each compound are shown in **Figure 1B**.

### PNS Protects TG-Induced ER Stress and Associated Cell Death

TG is a SERCA inhibitor, leading to depletion of ER calcium storage and decrease of the activity of Ca^2+^-dependent chaperones and thus resulting in an increase in unfolded proteins and an induction of UPR signaling (Denmeade and Isaacs, 2005). In this study, TG was used to induce ER stress.

PNS and TG exposure to H9c2 cells was conducted by MTT cell viability assay to set the concentration used in the following trials. PNS was first dissolved in DMSO, and then the serial dilutions of PNS (20, 40, 80, and 100 μg/ml) were tested with MTT assays and immunoblot of ER chaperone protein BiP expression. As shown in **Figure 1C**, PNS-treated cells exhibited no cytotoxic effect up to the highest concentration of 100 μg/ml. Furthermore, the effect of PNS on cell viability was shown in a concentration-dependent manner towards 1 μM TG-induced cell death, and cells pretreated with 40 μg/ml exhibited the best protection effects (**Figure 1C**), which corresponds to the downregulation of BiP expression by 40 μg/ml pretreatment (**Figure 1D**).

**Figure 2:**
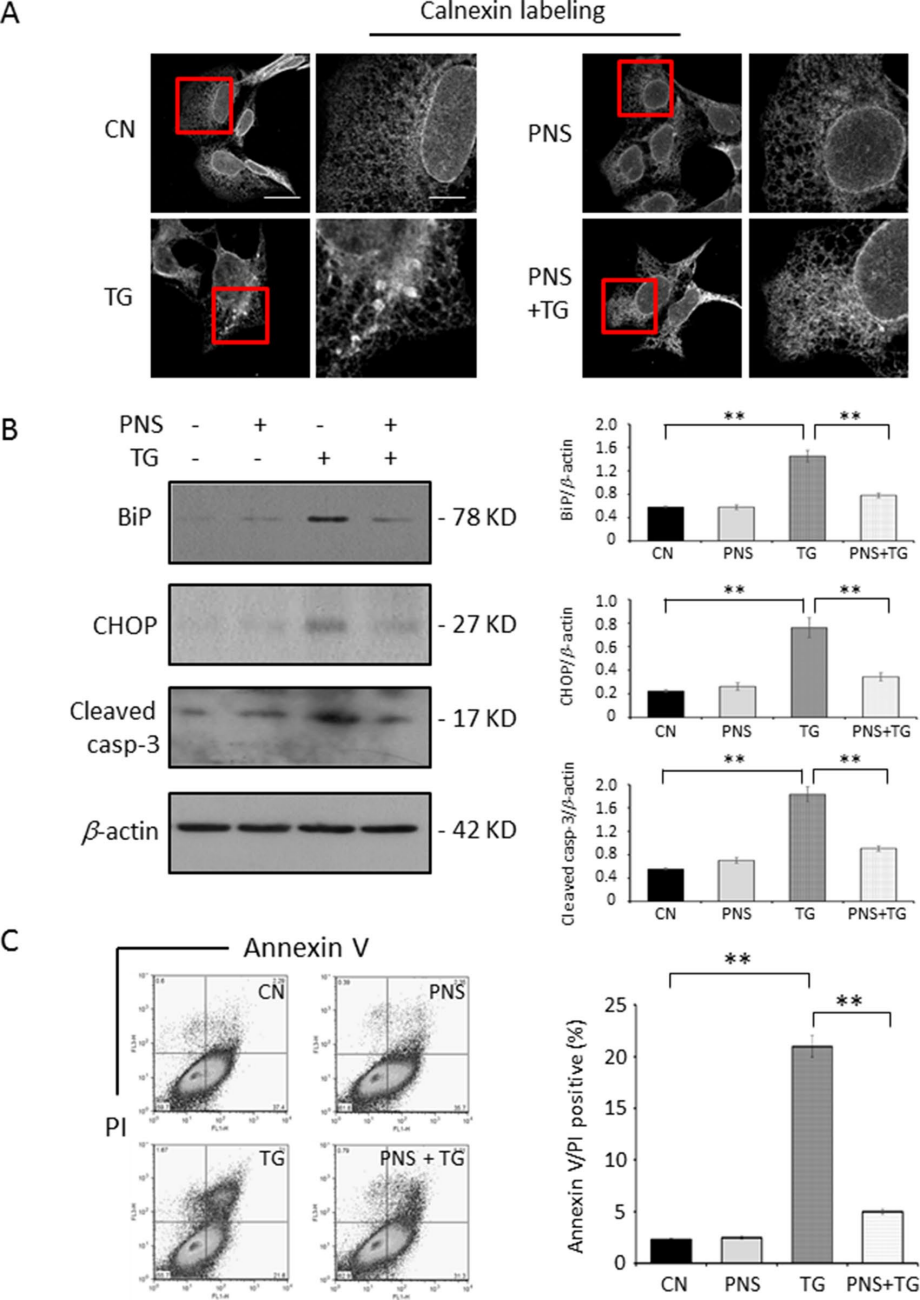
PNS prevents TG-induced ER stress response and cell apoptosis. **(A)** H9c2 cells, either untreated (CN group) or pretreated with 40 μg/ml PNS for 12 h (PNS group), before addition of 1 μM TG (TG group or PNS plus TG group) for 12 h were immunofluorescenced with primary anti-calnexin antibody. Scale bar, 30 μm; in box: 10 μm. **(B)** H9c2 cells treated as in A were immunoblotted with antibodies to BiP, CHOP, Cleaved caspase-3, and β-actin. Bands were quantified relative to β-actin by densitometry. **(C)** H9c2 cells treated as in A were double-stained with Annexin V/PI and analyzed by flow cytometry. Bar graph shows percentage of Annexin V/PI double-positive cells. (Mean ± SEM; **P < 0.01 relative to CN group or indicated group.) ER, endoplasmic reticulum; PI, propidium iodide; CHOP, the C/EBP homologous protein.

As shown in **Figure 2A**, the calnexin-labeled ER network in 1 μM TG-treated H9c2 cells for 12 h showed the disruption and condensation of the ER tubular network into large aggregates. However, when cells were pretreated with 40 μg/ml PNS for 12 h, TG no longer disrupted the ER tubular network. PNS treatment alone has no effect on the ER network morphology. TG increased the expression levels of BiP and CHOP as well as the apoptotic gene Cleaved caspase-3, while cells pretreated with PNS for 12 h attenuated a TG-induced upregulation of BiP, CHOP, and Cleaved caspase-3 expression (**Figure 2B**). Following this, we then investigated the effect of PNS prevention on TG-induced cell death. A 24 h TG treatment–induced cell death was further characterized by an Annexin V/PI double-staining *via* flow cytometry assay (**Figure 2C**). PNS pretreatment significantly reduced the number of Annexin V/PI double-positive cells induced by TG. These results demonstrate that PNS pretreatment promotes cardiac myocyte survival against TG-induced ER stress response and its associated cell death.

### PNS Suppresses Intracellular Ca^2+^ Homeostasis

Aberrant Ca^2+^ regulation in ER results in protein unfolding, due to the Ca^2+^-dependent nature of ER chaperone proteins such as BiP and calreticulin (Ma and Hendershot, 2004). Ca^2+^ is also a key regulator of cell death and survival. We therefore investigated the effects of PNS on ER stress–induced cytosolic, mitochondria, as well as ER Ca^2+^ homeostasis.

**Figure 3:**
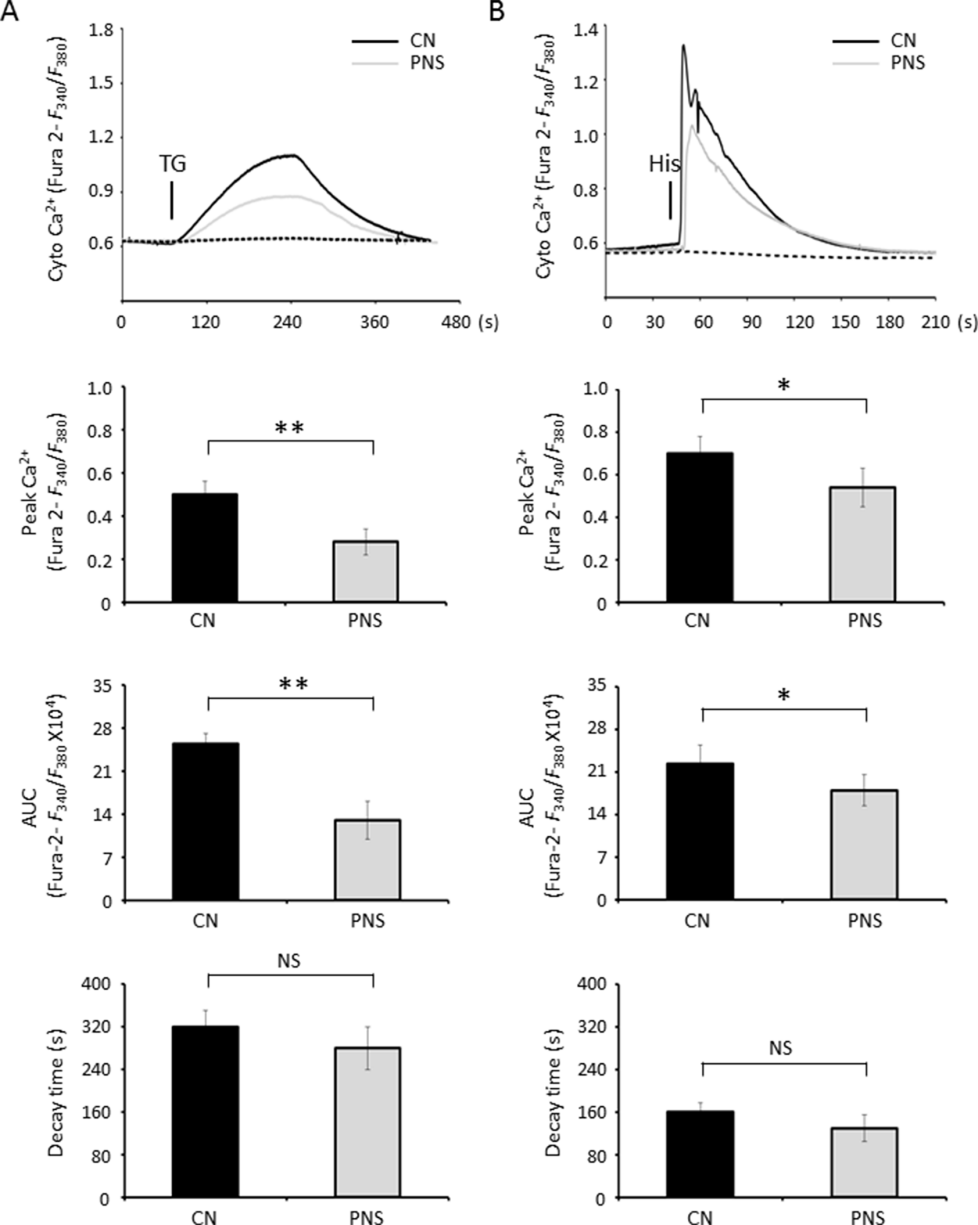
PNS suppresses cytosolic Ca^2+^ transients evoked by TG and histamine. **(A)** Representative recordings of TG-evoked cytosolic Ca^2+^ transients recorded by Fura-2 ratios (*F*_340_/*F*_380_) in H9c2 cells with (grey, PNS group) or without (dark, CN group) PNS pretreatment (40 μg/ml; 12 h), as indicated. Bar graphs show cytosolic Ca^2+^ peak amplitude, area under the curve (AUC), as well as time decay of Ca^2+^ transient response to TG stimulation. **(B)** Representative recordings of histamine-evoked cytosolic Ca^2+^ transients recorded by Fura-2 ratios (*F*_340_/*F*_380_) in H9c2 cells with (grey) or without (dark) PNS pretreatment (40 μg/ml; 12 h), as indicated. Bar graphs show cytosolic Ca^2+^ peak amplitude, AUC, as well as time decay of Ca^2+^ transient response to histamine stimulation. (Mean ± SEM; 60–80 responding cells; NS, not significant; *P < 0.05, **P < 0.01 relative to CN group.)

Cytosolic Ca^2+^ was measured by calcium imaging of the ratiometric Ca^2+^ indicator, Fura-2 intensity ratio (*F*_340_/*F*_380_). After loading with 5 μM Fura-2 AM, cardiac myocytes were subjected to TG stimulation during the acquisition. In response to TG, a significant Ca^2+^ transient appeared in untreated cells, while PNS pretreatment attenuated peak amplitude and area under the curve (AUC) of TG-induced cytosolic Ca^2+^ transients. PNS pretreatment did not show a significant effect on cytosolic Ca^2+^ fluorescence decay (**Figure 3A**). Next, we determined whether PNS pretreatment affected inositol triphosphate (IP_3_) receptor (IP_3_R)–mediated or RyR-mediated ER Ca^2+^ release using histamine stimulation. Again, PNS pretreatment attenuated peak amplitude and AUC of histamine-induced cytosolic Ca^2+^ transients without affecting histamine-induced cytosolic Ca^2+^ fluorescence decay (**Figure 3B**). However, there were no significant differences in the basal *F*_340_/*F*_380_ ratios detected between untreated and PNS-pretreated cells, suggesting that PNS pretreatment does not affect the intracellular Ca^2+^ concentration.

**Figure 4:**
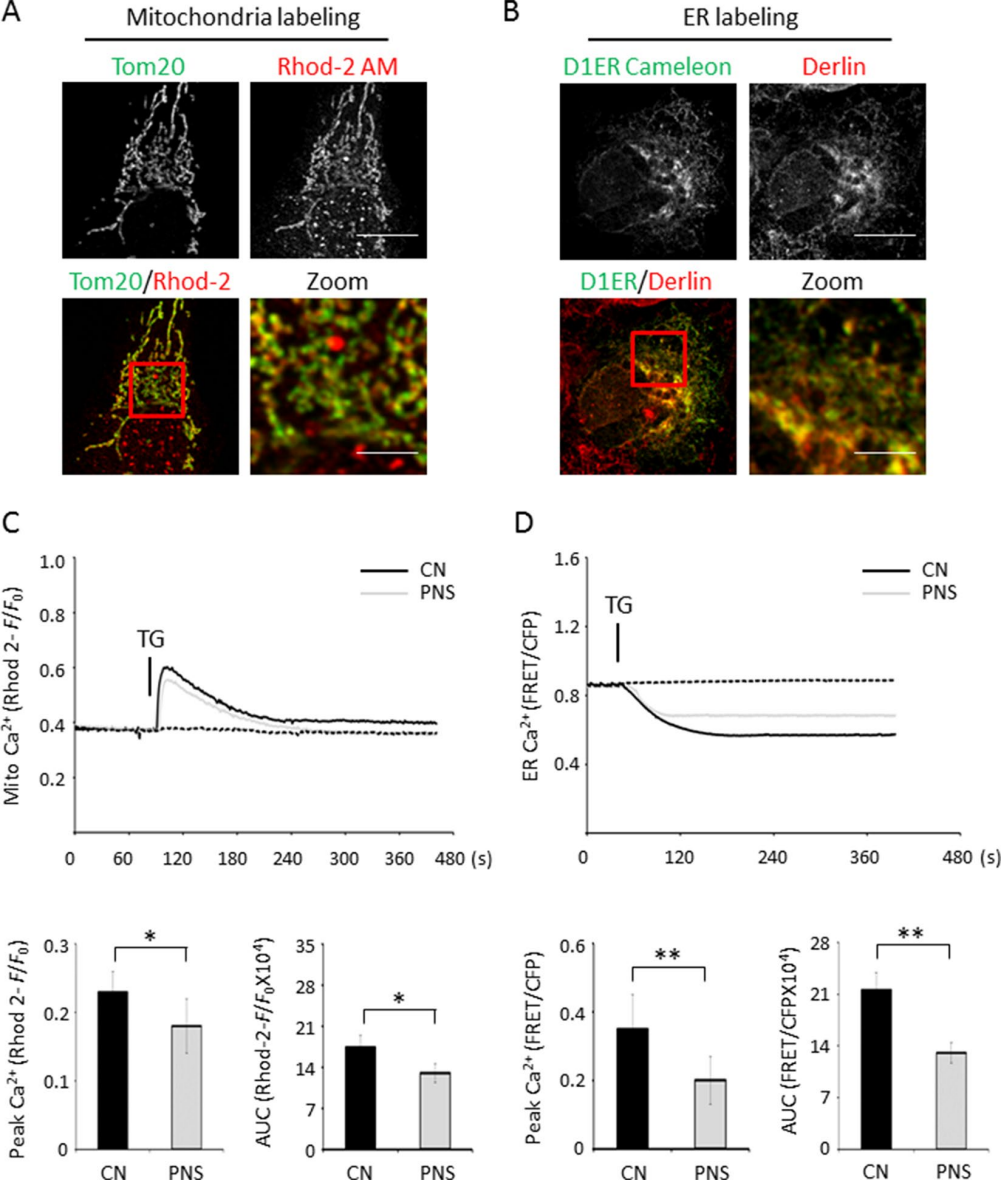
PNS suppresses mitochondrial Ca^2+^ uptake and ER Ca^2+^ release induced by TG. **(A)** Confocal microscope images of Rhod-2 AM loaded H9c2 cells counterstained with anti-Tom20 mitochondrial antibody. Scale bar, 10 μm; in box: 3 μm. **(B)** Confocal microscope images of H9c2 cells loaded with D1ER cameleon and anti-derlin antibody. Scale bar, 10 μm; in box: 3 μm. **(C)** Representative recordings of TG-evoked mitochondrial Ca^2+^ elevation recorded by Rhod-2 fluorescence (*F*/*F*_0_) in H9c2 cells with (grey, PNS group) or without (dark, CN group) PNS pretreatment (40 μg/ml; 12 h), as indicated. Bar graphs show mitochondrial Ca^2+^ peak amplitude and AUC in response to TG stimulation. (Mean ± SEM; 60–80 responding cells; *P < 0.05, **P < 0.01 relative to CN group.) **(D)** Representative recordings of TG-induced ER Ca^2+^ dynamics were recorded by the FRET-to-CFP emission ratio (FRET/CFP) in H9c2 cells with (grey, PNS group) or without (dark, CN group) PNS pretreatment (40 μg/ml; 12 h), as indicated. Bar graphs show ER Ca^2+^ peak amplitude and AUC in response to TG stimulation. (Mean ± SEM; 20–30 responding cells; *P < 0.05, **P < 0.01 relative to CN group.) FRET, fluorescence resonance energy transfer; AM, acetoxymethyl; D1ER, ER-targeted cameleon; CFP, cyan fluorescent protein.

ER stress–induced mitochondrial and ER Ca^2+^ dynamics were investigated using the mitochondrial Ca^2+^ reporter Rhod-2 AM and the ER Ca^2+^ reporter D1ER cameleon, respectively. Confocal fluorescence images of Rhod-2 AM and anti-Tom20 labeling, as well as anti-derlin and D1ER cameleon labeling in H9c2 cells, showed essentially high colocalization of Rhod-2 and mitochondria as well as D1ER cameleon and ER, respectively (**Figures 4A, B**). Mitochondrial Ca^2+^ dynamics showed that PNS pretreatment reduced the elevation of mitochondrial Ca^2+^ uptake induced by TG stimulation (**Figure 4C**, peak amplitude and AUC). However, no significant differences in the basal *F/F*_0_ ratios were detected between untreated and PNS-pretreated cells, suggesting that PNS pretreatment does not affect the relative mitochondrial Ca^2+^ concentration. In addition, acute TG stimulation induced a time-resolved reduction of ER calcium concentration. TG-induced ER Ca^2+^ release was significantly reduced in PNS-pretreated cells compared with those in untreated cells (**Figure 4D**, peak amplitude and AUC). However, no significant differences in the basal cameleon FRET ratios were detected between untreated and PNS-pretreated cells, suggesting that PNS does not affect ER Ca^2+^ content. The results of mitochondrial Ca^2+^ and ER Ca^2+^ dynamics indicate that PNS pretreatment reduces mitochondrial Ca^2+^ uptake and ER Ca^2+^ release upon TG stimulation, consistent with the effect of PNS on the reduction of TG or histamine-evoked cytosolic Ca^2+^ transients.

### PNS Prevention of TG-Induced ER Stress and Associated Cell Apoptosis Is Ca^2+^ Dependent

Previous data have shown that PNS suppressed the intracellular Ca^2+^ homeostasis, suggesting that intracellular calcium may be involved in the PNS prevention of the ER stress response and its associated apoptotic events. We then applied Bapta-AM and ionomycin plus Ca^2+^ to adjust the intracellular Ca^2+^ concentration. Bapta, an intracellular Ca^2+^ chelator, induces cytosolic Ca^2+^ decay, while ionomycin plus Ca^2+^ induces cytosolic Ca^2+^ accumulation. We therefore investigated the effect of intracellular Ca^2+^ reduction and induction on PNS protection in TG-induced ER stress and the associated apoptosis.

**Figure 5:**
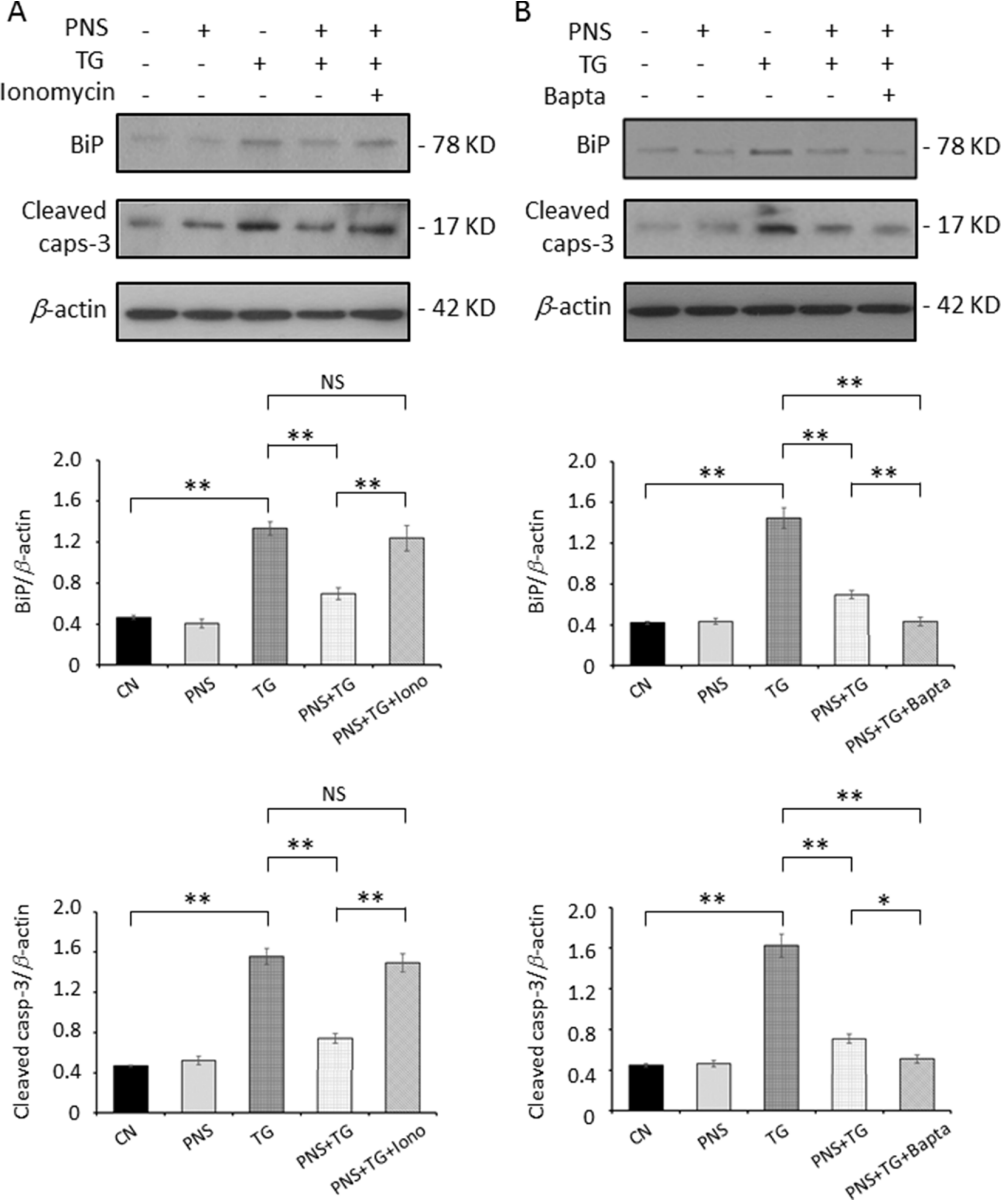
PNS prevention of TG-induced ER stress and apoptosis is Ca^2+^ dependent. H9c2 cells were either untreated (CN group) or pretreated with 40 μg/ml PNS for 12 h (PNS group) before addition of 1 μM TG for 12 h in the presence of 1 mM Ca^2+^ plus 1 μM ionomycin (Iono; **A**) or 40 μM Bapta-AM (Bapta; **B**), as indicated. Treated cells were immunoblotted with antibodies to BiP, Cleaved caspase-3, as well as β-actin, and bands were quantified relative to β-actin by densitometry. (Mean ± SEM; NS, not significant; **P < 0.01 relative to CN group or indicated group.)

Cells were treated with 1 μM ionomycin plus 1 mM extracellular Ca^2+^, which resulted in an increase of cytosolic Ca^2+^. TG-induced upregulation of BiP and Cleaved caspase-3 was no longer attenuated by PNS pretreatment (**Figure 5A**). However, treatment of cells with Bapta enhanced the downregulation effect of PNS on TG-induced BiP and Cleaved caspase-3 expression (**Figure 5B**). These results confirmed that Ca^2+^ plays a critical role in the ER stress response and the associated cell apoptosis, and PNS prevention of ER stress and promotion of cell survival are Ca^2+^ dependent.

### RyR_2_ Mediates PNS Protection Against the ER Stress Response and Apoptosis

**Figure 6:**
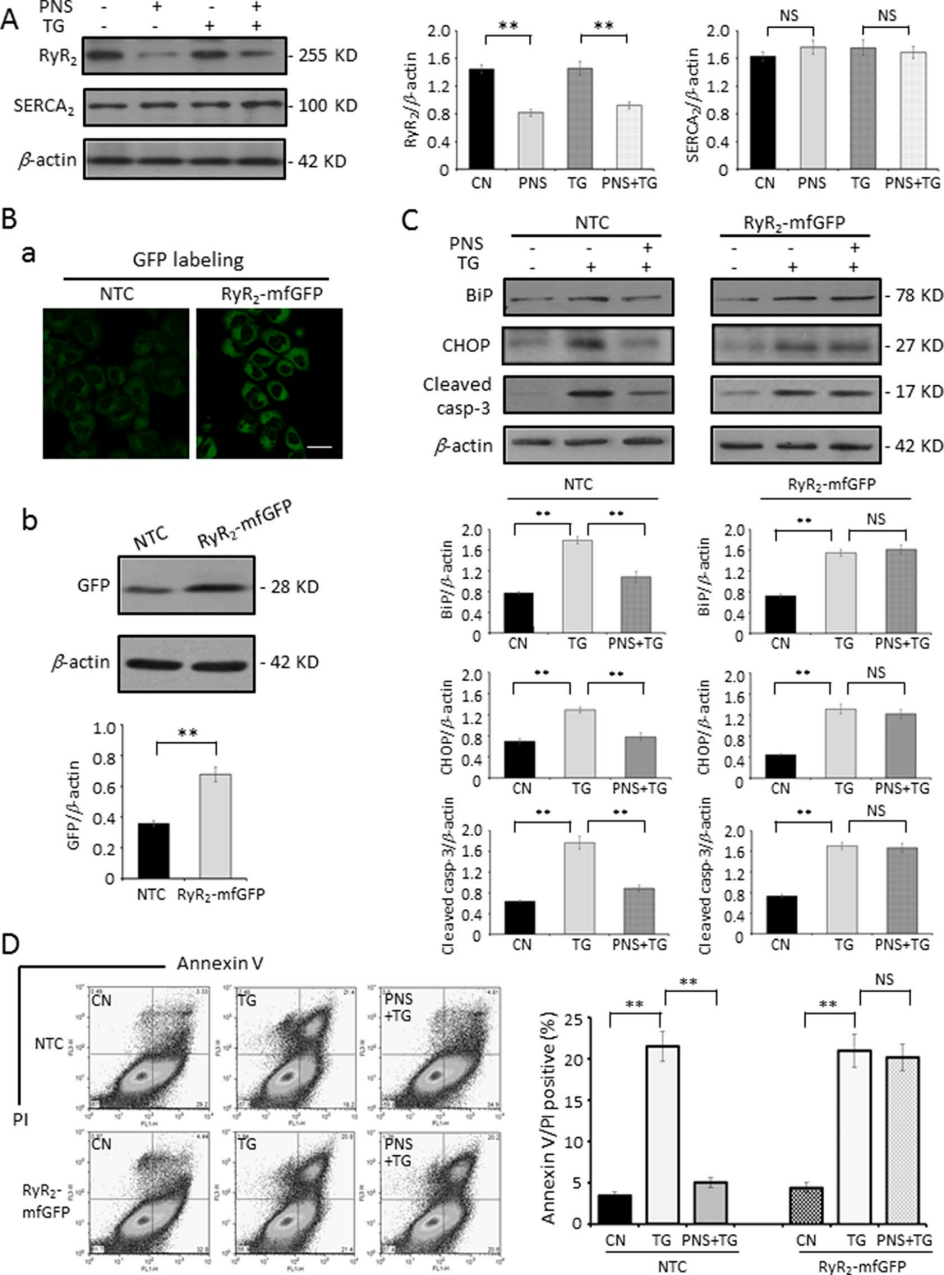
PNS prevention of TG-induced ER stress and apoptosis is regulated by RyR_2_. **(A)** H9c2 cells, either untreated (CN group) or pretreated with 40 μg/ml PNS for 12 h (PNS group) before addition of 1 μM TG (TG group or PNS plus TG group) for 12 h, were immunoblotted to antibodies to RyR_2_ and SERCA_2_ as well as β-actin. Bands were quantified relative to β-actin by densitometry. **(B)** Non-target control (NTC group) or RyR_2_-mfGFP (RyR_2_-mfGFP group) inducible stable HL-1 cell lines were identified by immunofluorescence and immunoblot to anti-GFP antibody. Scale bar, 50 μm. **(C)** Non-target control (NTC group) or RyR_2_-mfGFP transfected (RyR_2_-mfGFP group) HL-1 cells either untreated (CN group) or pretreated with 40 μg/ml PNS for 12 h before addition of 1 μM TG for 12 h (TG group or PNS plus TG group) were immunoblotted with antibodies to BiP, CHOP, and Cleaved caspase-3 as well as β-actin. Bands were quantified relative to β-actin by densitometry. **(D)** NTC or RyR_2_-mfGFP HL-1 cells treated as indicated in **(C)** were double-stained with Annexin V and PI, and analyzed by flow cytometry. Bar graph shows percentage of Annexin V/PI double-positive cells. (Mean ± SEM; NS, not significant; **P < 0.01 relative to CN group or indicated group.) RyR_2_, ryanodine receptor type-2. SERCA_2_, sarco/endoplasmic reticulum Ca^2+^-ATPase; mfGFP; multifunctional GFP.

The SR/ER calcium ATPase (SERCA) is responsible for transporting Ca^2+^ from the cytosol into the lumen of the SR following muscular contraction. The Ca^2+^ sequestering activity of SERCA facilitates muscular relaxation in both cardiac and skeletal muscle (Lytton et al., 1991). Release of Ca^2+^ from the ER is critical in the cellular signaling mediated by second messengers, such as IP_3_, cytosolic adenosine diphosphate ribose (ADP-ribose), and other regulators, *via* effects on IP_3_Rs or RyRs (Berridge et al 2000; Benkusky et al., 2004). Next, we investigated the effects of PNS treatment on ER Ca^2+^ handling proteins, such as SERCA_2_ and RyR_2_, in H9c2 cells. **Figure 6A** showed that PNS pretreatment significantly decreased the RyR_2_ expression, which is consistent with the results that PNS pretreatment significantly attenuates peak amplitude and AUC of histamine-induced cytosolic Ca^2+^ transients (**Figure 3B**) as well as dramatically attenuates ER Ca^2+^ release (**Figure 4D**). However, PNS did not show significant effects on SERCA_2_ expression (**Figure 6A**).

To determine the role of RyR_2_ in PNS prevention of ER stress response and the associated apoptosis, we generated a RyR_2_-mfGFP stably transfected HL-1 cell line, which showed a 180–200% increase in RyR_2_ expression compared with NTC transfected HL-1 cells by immunofluorescence (a) and immunoblot (b) analyses, respectively (**Figure 6B**). The same as observed in H9c2 cells, PNS pretreatment decreased TG-induced upregulation of BiP, CHOP, and Cleaved caspase-3 expression in NTC HL-1 cells. However, in RyR_2_-mfGFP stably transfected cells, PNS pretreatment revealed no significant effect on the TG-induced upregulation of BiP, CHOP, and Cleaved caspase-3 expression (**Figure 6C**). Again, Annexin V/PI double-staining analyses by flow cytometry showed that PNS pretreatment significantly reduced the number of Annexin V/PI double-positive cells induced by TG stimulation in NTC HL-1 cells, while having no protection effects on RyR_2_-mfGFP stably transfected HL-1 cells (**Figure 6D**). These results suggest that PNS protection against ER stress and its associated apoptosis is through RyR_2_-mediated ER Ca^2+^ release.

**Figure 7:**
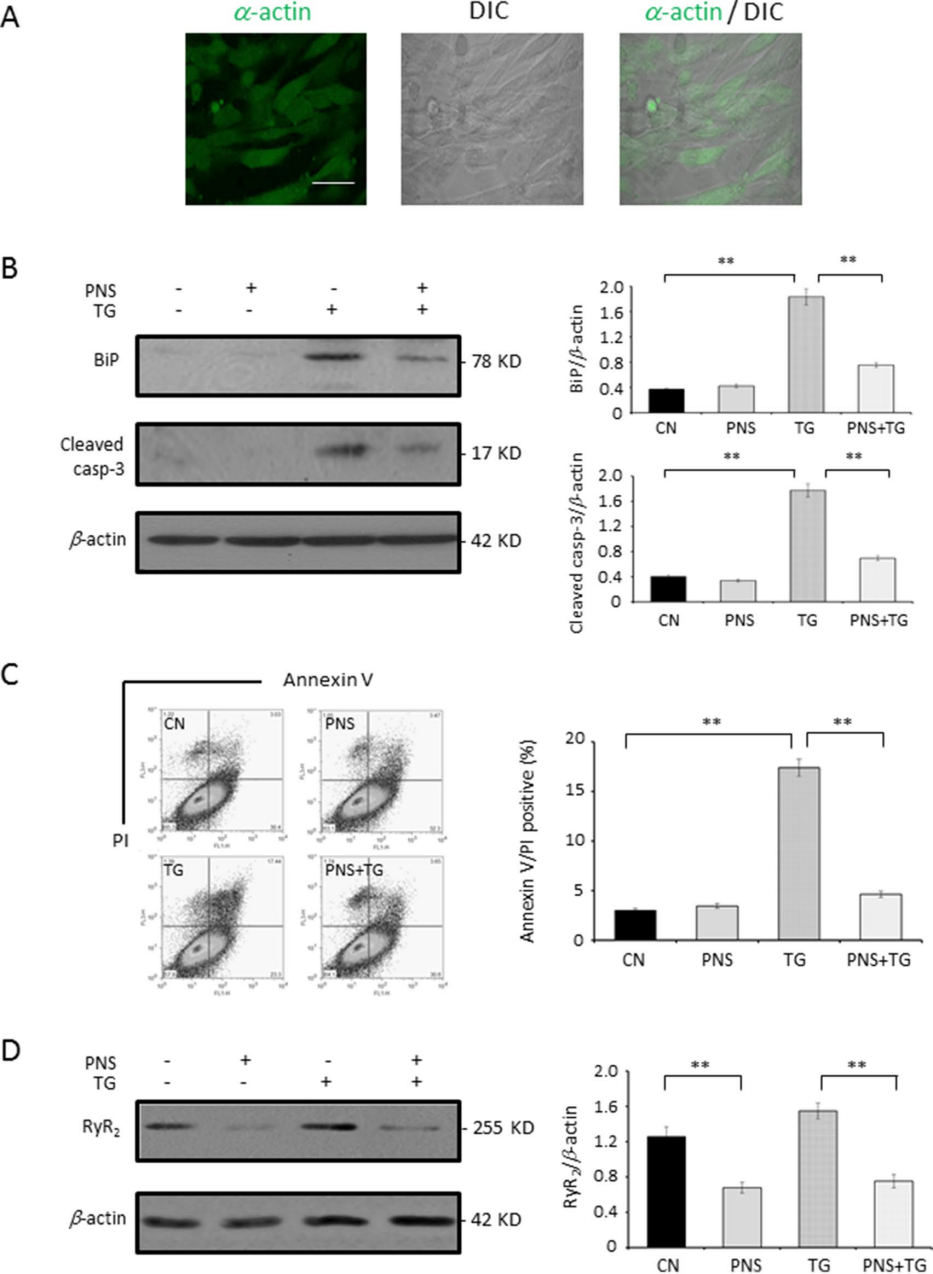
PNS prevents TG-induced ER stress response and cell apoptosis in primary cultured cardiomyocytes. **(A)** Primary cultured cardiomyocytes were immunofluorescenced with primary anti-*α*-actin antibody. Scale bar, 50 μm. **(B)** Primary cultured cardiomyocytes either untreated (CN group) or pretreated with 40 μg/ml PNS for 12 h (PNS group), before addition of 1 μM TG (TG group or PNS plus TG group), were immunoblotted with antibodies to BiP and Cleaved caspase-3 and β-actin. Bands were quantified relative to β-actin by densitometry. **(C)** Primary cultured cardiomyocytes treated as in **(B)** were double-stained with Annexin V/PI and analyzed by flow cytometry. Bar graph shows percentage of Annexin V/PI double-positive cells. **(D)** Primary cultured cardiomyocytes treated as in **(B)** were immunoblotted with antibodies to RyR_2_ and β-actin. Bands were quantified relative to β-actin by densitometry. (Mean ± SEM; **P < 0.01 relative to CN group or indicated group.)

The protective effect of PNS in TG-induced ER stress response and the associated cell death as well as the RyR_2_ expression was then verified in primary cultured neonatal rat cardiomyocytes. As shown in **Figure 7A**, the confocal images showed that more than 90% of cells were identified as *α*-actin fluorescence positive and therefore confirmed that the purification of cardiomyocytes was over 90%. PNS pretreatment revealed a significant effect of reversing TG-induced upregulation of BiP and Cleaved caspase-3 expression (**Figure 7B**). The Annexin V/PI double-staining analysis by flow cytometry showed that PNS pretreatment significantly reduced the number of Annexin V/PI double-positive cells induced by TG stimulation (**Figure 7C**). Interestingly, PNS pretreatment significantly decreased the RyR_2_ expression in primary cultured cardiomyocytes as well (**Figure 7D**), which is consistent with the results of H9c2 cells (**Figure 6A**). These results suggest that PNS is effective in protecting both established and primary cultured cardiomyocytes from TG-induced ER stress and its associated cell death mediated by RyR_2_.

Taken together, these results indicate that PNS has significant protective effects in TG-induced ER stress and its associated cell death in cardiac myocytes, while its prevention effect is Ca^2+^ dependent. PNS has a significant suppression effect on intracellular Ca^2+^ hemostasis such as cytosolic Ca^2+^, mitochondria Ca^2+^, as well as ER Ca^2+^ release upon TG stimulation, and this activity is dependent on the expression of RyR_2_, suggesting that PNS prevention of cardiac myocytes towards ER stress and its associated cell death is regulated by RyR_2_-mediated ER Ca^2+^ release.

## Discussion

Numerous studies have shown that cardiac myocytes are vulnerable to cellular ER stress and contribute to the pathogenesis of several cardiovascular derangements through exposure to hyperoxidation, inflammation, apoptosis, etc. These findings have sparked interest, demonstrating a link between ER stress and cardiovascular pathogenesis, while the elevation of ER stress–associated apoptosis has been proposed to contribute to various cardiovascular diseases (Camargo et al., 2018; Chang et al., 2018; Huang et al., 2018). Hence, modulation of ER stress, especially downstream of calcium-mediated apoptotic execution pathways, becomes critical in understanding the mechanism and the development of a novel target of pathogenesis of cardiovascular diseases.

*Panax notoginseng saponins* are the main active ingredients of *Panax notoginseng*, which are derived from the rhizomes of Araliaceae plant *Panax notoginseng*. Over the past 40 years, numerous researchers have devoted their efforts to confirming the effectiveness of PNS in cardiovascular diseases and strokes (Liu et al., 2014; Duan et al., 2017). Presently, PNS is available as an over-the-counter drug both in China and worldwide. Furthermore, many *in vitro* experiments have shown that PNS could regulate lipid metabolism and inflammation, reduce myocardial damage, attenuate cardiomyocyte apoptosis, and inhibit platelet adhesion to injured endothelial cells (Fan et al., 2012; Wang et al., 2016; Zhou et al., 2018). However, the effect of PNS on ER stress and the associated cell death in cardiac myocytes has not been reported.

**Figure 8:**
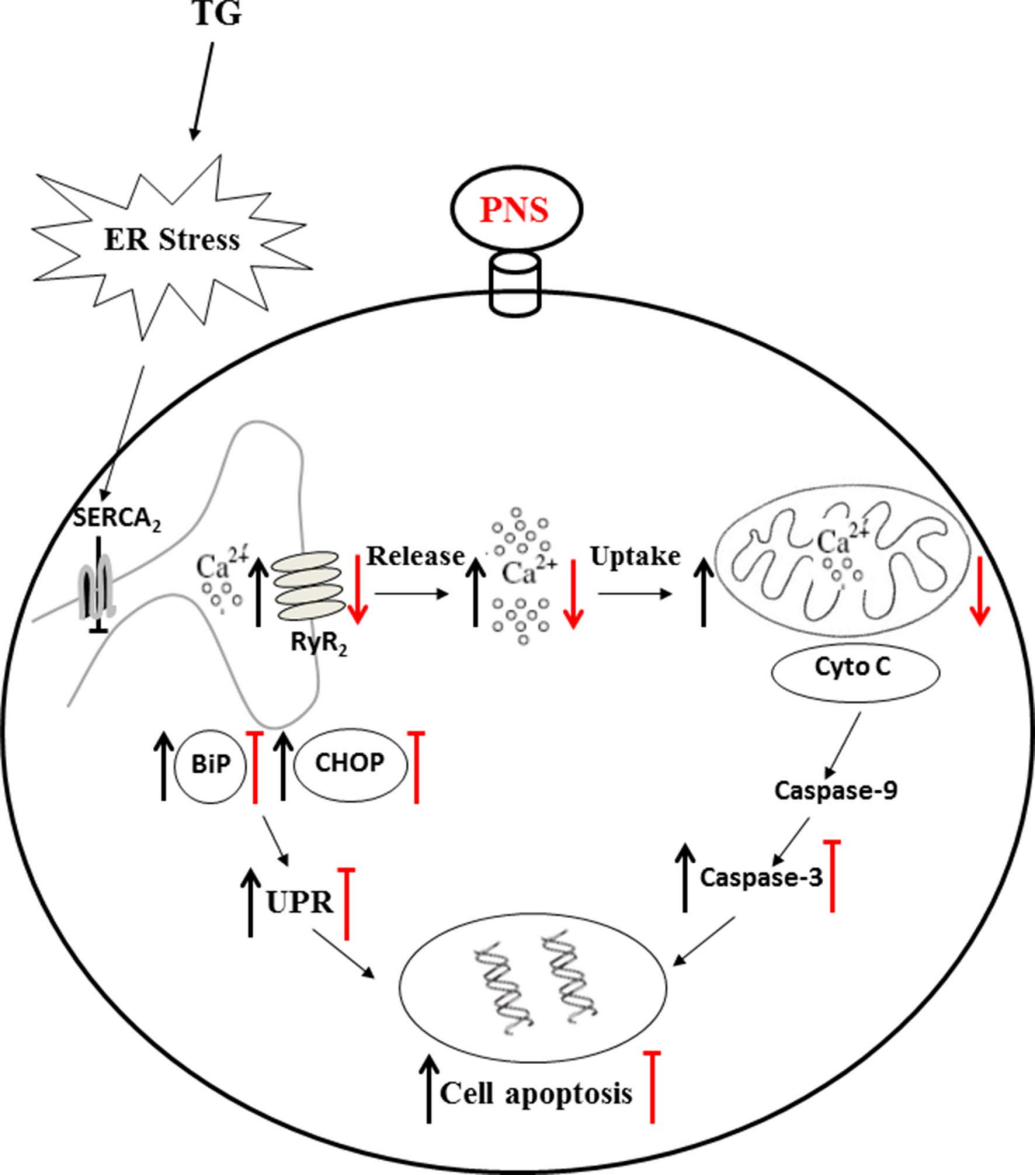
Mechanism of PNS protects ER stress–induced cell death. TG inhibits Ca^2+^ re-uptake from ER to cytosol through SERCA_2_ and thereby elevates intracellular concentration and mitochondrial calcium uptake. TG disturbs the calcium homeostasis and causes protein-folding dysfunction, and thus, the accumulated misfolded/unfolded proteins (e.g. BiP and CHOP) induce ER stress. Prolonged TG treatment initiates the intrinsic apoptotic pathway by permeabilizing the mitochondrial membrane and thus releasing cytochrome c and AIF to cytosol, resulting in apoptosome formation and leading to activation of caspase-3. PNS pretreatment significantly reduced upregulation of BiP, CHOP, and Cleaved caspase-3 induced by TG. PNS reduced the elevation of cytosolic calcium transients, mitochondrial calcium uptake, as well as ER calcium release in response to either TG or histamine. PNS decreases the RyR_2_ expression, and PNS prevention of ER stress and associated apoptosis is mediated by RyR_2_ expression. These results suggest that by the intermediary through regulation of intracellular calcium homeostasis, especially suppression of ER calcium release mediated by RyR_2_ and thus inhibition of the cytosolic and mitochondrial calcium overload, PNS therefore protected against ER stress–induced cell death. AIF, apoptosis inducing factor.

The data presented in the current study provide insights into the exploration of the critical mechanism of PNS prevention of ER stress and the associated cell death in cardiac myocytes. Firstly, we have demonstrated here that PNS significantly protects against TG-induced ER stress and its associated apoptosis. Secondly, PNS reduced the elevation of cytosolic calcium transients, mitochondrial calcium uptake, as well as ER calcium release in response to either TG or histamine. Lastly, PNS protection in TG-induced ER stress and cell apoptosis is Ca^2+^ dependent. In addition, PNS prevention of ER stress and cell apoptosis is mediated by decreasing the RyR_2_ expression. Therefore, PNS is identified as a novel potential treatment against cardiac myocyte death towards cell ER stress (**Figure 8**).

Calcium plays crucial roles in ER stress and cell death. Sustained elevation of cytosolic Ca^2+^ released from the ER results in cytosolic Ca^2+^ overload. Moreover, the interactions between the ER and mitochondria facilitate the transfer of Ca^2+^ between these two organelles, which represents important mechanisms of apoptosis regulation (Krebs et al., 2015). The downstream effectors of Ca^2+^-induced cell death are also due to the induction of mitochondrial permeability transition, which is induced upon entry of excessive amounts of Ca^2+^ into the matrix of mitochondria (Bernardi, 1999; Kroemer and Reed, 2000).

We then investigated the cytosolic, mitochondrial, as well as ER Ca^2+^ dynamics in response to ER stressor TG stimulation. As result, PNS pretreatment significantly reduced the peak amplitude and total AUC elevation of cytosolic Ca^2+^ without affecting the basal cytosolic Ca^2+^ concentration. More importantly, PNS pretreatment also attenuated the Ca^2+^ uptake by mitochondria without affecting the relative mitochondrial Ca^2+^ concentration, suggesting that PNS regulates cytosolic and mitochondrial Ca^2+^ overload upon TG and histamine stimulation, which may be through reducing the ER Ca^2+^ release. We then tested the histamine-induced cytosolic Ca^2+^ transients as well as TG-induced ER calcium release dynamics. Again, these results show that PNS pretreatment attenuated the peak amplitude of Ca^2+^ release and total AUC of the cytosolic Ca^2+^ transients evoked by histamine and TG-induced ER Ca^2+^ release, suggesting that PNS-regulated intracellular Ca^2+^ homeostasis is mediated by ER Ca^2+^ release.

To test whether the induction and reduction of cytosolic Ca^2+^ affects the prevention effect of PNS in ER stress response and cell apoptosis, we first treated cells with cell-permeable Ca^2+^ chelator, Bapta-AM (Fu et al., 2011), and showed that PNS significantly reduced the expression of BiP, CHOP, and apoptotic gene Cleaved caspase-3 in response to TG. However, cells were incubated with extracellular Ca^2+^ containing Ca^2+^ ionophore ionomycin to induce elevation of cytosolic Ca^2+^, which prevents PNS effects on protection against TG-induced ER stress and cell death. This demonstrates that the reduction or elevation of the intracellular Ca^2+^ plays important roles in PNS protection against ER stress response and cell apoptosis.

The cardiac RyR_2_ plays an important role in the cardiac physiology by regulating the Ca^2+^ release from the SR (Fu et al., 2008; Belevych et al., 2013). Expression of the RyR_2_ is significantly decreased by PNS pretreatment, suggesting that PNS has effects on RyR_2_ expression and thereby regulating ER Ca^2+^ release. This may be a critical element that affects the intracellular Ca^2+^ homeostasis and mediates PNS pro-survival activity. Next, the RyR_2_ overexpression cell line was generated to verify whether the RyR_2_ expression affects the PNS protection in ER stress and its associated apoptosis. In RyR_2_-overexpressed HL-1 cells, PNS pretreatment no longer prevents TG-induced ER stress and its associated cell apoptosis, indicating that PNS prevention of TG-induced ER stress response and associated cell death is mediated by RyR_2_. Finally, PNS protective effects in TG-induced ER stress and associated cell death as well as downregulation of RyR_2_ expression were verified in the primary cultured neonatal cardiomyocytes.

In the present study, we identified the signaling regulatory pathway of PNS protection in ER stress–induced cell death in cardiac myocytes. Our results characterized that PNS pretreatment significantly reduced ER stress response and its associated cell apoptosis. PNS reduced the elevation of cytosolic calcium transients, mitochondrial calcium uptake, as well as ER calcium release. PNS prevention of ER stress and cell apoptosis is mediated by RyR_2_ expression. These results suggest that PNS protects cardiac myocytes against TG-induced ER stress and its associated cell apoptosis through the intermediary regulation of intracellular calcium homeostasis—the suppression of ER calcium release mediated by RyR_2_—and thus the inhibition of the cytosolic and mitochondrial calcium overload. Regulation of ER Ca^2+^ release by PNS defines a novel mechanism for the natural product medicine in the regulation of ER stress response. PNS interaction with RyR_2_-mediated ER Ca^2+^ release may therefore contribute to the positive response of cardiac myocytes to intracellular ER stress and its associated cell death.

## Data Availability

The datasets generated for this study are available on request to the corresponding author.

## Ethics Statement

The experimental protocol for using animals in this research was approved by the Ethics Committee for Scientific Research and Clinical Trials of the First Affiliated Hospital of Zhengzhou University (2018-KY-96).

## Author Contributions

JC, RX, LiL, LLX, and JS carried out the experiments, WZ and XB analyzed the data, and GL and LL designed the study and wrote the manuscript. All authors read and approved the final manuscript.

## Funding

This work was supported by a grant from National Natural Science Foundation of China (U1304804).

## Conflict of Interest Statement

The authors declare that the research was conducted in the absence of any commercial or financial relationships that could be construed as a potential conflict of interest.

## Abbreviations

PNS, *Panax notoginseng* saponin; ER, endoplasmic reticulum; HPLC, high-performance liquid chromatograph; MTT, methyl thiazolyl tetrazolium; TG, thapsigargin; UPR, unfolded protein response; PI, propidium iodide; RyR_2_, ryanodine receptor type-2; IP3R, 1,4,5-trisphosphate receptors; SERCA, sarco/endoplasmic reticulum Ca^2^+-ATPase; AUC, area under the curve; FRET, fluorescence resonance energy transfer; NTC, non-targeted control.
